# Tobacco Cutworm (*Spodoptera Litura*) Larvae Silenced in the NADPH-Cytochrome P450 Reductase Gene Show Increased Susceptibility to Phoxim

**DOI:** 10.3390/ijms20153839

**Published:** 2019-08-06

**Authors:** Hong-Yi Ji, Christian Staehelin, Yan-Ping Jiang, Shi-Wei Liu, Zhi-Hui Ma, Yi-Juan Su, Jia-En Zhang, Rui-Long Wang

**Affiliations:** 1 Guangdong Province Key Laboratory of Eco-Circular Agriculture, South China Agricultural University, Guangzhou 510642, China; 2 Key Laboratory of Agro-Environment in the Tropics, Ministry of Agriculture, South China Agricultural University, Guangzhou 510642, China; 3 State Key Laboratory of Biocontrol and Guangdong Key Laboratory of Plant Resources, School of Life Sciences, Sun Yat-sen University, East Campus, Guangzhou 510006, China; 4 New Rural Development Research Institute of South China Agricultural University, Guangzhou 510642, China

**Keywords:** *Spodoptera litura*, NADPH-cytochrome, P450 reductase, RNAi, insecticide susceptibility, phoxim

## Abstract

Nicotinamide adenine dinucleotide phosphate (NADPH)-cytochrome P450 reductases (CPRs) function as redox partners of cytochrome P450 monooxygenases (P450s). CPRs and P450s in insects have been found to participate in insecticide resistance. However, the CPR of the moth *Spodoptera litura* has not been well characterized yet. Based on previously obtained transcriptome information, a full-length CPR cDNA of *S. litura* (*SlCPR*) was PCR-cloned. The deduced amino acid sequence contains domains and residues predicted to be essential for CPR function. Phylogenetic analysis with insect CPR amino acid sequences showed that *SlCPR* is closely related to CPRs of Lepidoptera. Quantitative reverse transcriptase PCR (RT-qPCR) was used to determine expression levels of *SlCPR* in different developmental stages and tissues of *S. litura*. *SlCPR* expression was strongest at the sixth-instar larvae stage and fifth-instar larvae showed highest expression in the midgut. Expression of *SlCPR* in the midgut and fat body was strongly upregulated when fifth-instar larvae were exposed to phoxim at LC_15_ (4 μg/mL) and LC_50_ (20 μg/mL) doses. RNA interference (RNAi) mediated silencing of *SlCPR* increased larval mortality by 34.6% (LC_15_ dose) and 53.5% (LC_50_ dose). Our results provide key information on the *SlCPR* gene and indicate that *SlCPR* expression levels in *S. litura* larvae influence their susceptibility to phoxim and possibly other insecticides.

## 1. Introduction

The tobacco cutworm, *Spodoptera litura* (F.) (Lepidoptera, Noctuidae) is a serious polyphagous insect pest. The moth has a broad host range that includes economically important crops such as tomato, cotton, and groundnut [1]. Over recent years, many field populations of *S. litura* acquired resistance to various insecticides, particularly in Pakistan, China, and India. The task of controlling insecticide-resistant *S. litura* populations is becoming exceedingly challenging [1,2,3]. Phoxim has become one of the most widely used organophosphate insecticides for the control of *S. litura* [1,2,3].

Cytochrome P450 monooxygenases (CYPs or P450s) belong to a superfamily of heme-containing enzymes that catalyze the monooxygenation of xenobiotics and endogenous compounds [4,5]. Insect P450s can metabolize and detoxify insecticides and thus play an important role in evolution of insecticide resistance [4,5]. Various studies suggested that expression of specific P450s is induced when insects are exposed to insecticides. For example, our previous work on *S. litura* P450s suggested that insecticide-induced *CYP9A40* [6] and *CYP321B1* [7] play an important role in insecticide detoxification. Similarly, transcript levels of *CYP49A1*, *CYP6AB4*, *CYP9A19*, and *CYP9A22* in the fat body of *Bombyx mori* were expressed at high levels after 24, 48, and 72 h of phoxim treatment, suggesting that P450 genes expressed in the fat body are associated with detoxification of phoxim [8].

Ncotinamide adenine dinucleotide phosphate (NADPH)-cytochrome P450 reductases (CPRs), important redox partners of P450s, play a crucial role in providing electrons from nicotinamide adenine dinucleotide phosphate (NADPH) to P450s via two flavin cofactors [9,10]. CPRs belong to the electron transfer flavoprotein family whose members contain conserved binding domains to NADP, the flavin mononucleotide (FMN) cofactor, and the flavin adenine dinucleotide (FAD) cofactor [11,12]. CPR genes have been identified in various insect species such as *S. litura* [13], *Spodoptera littoralis* [14], *Cimex lectularius* [15], *Helicoverpa armigera* [16], *Nilaparvata lugens* [17], *Bactrocera dorsalis* [18], *Spodoptera exigua* [19], *Laodelphax striatellus* [12], *Cnaphalocrocis medinalis* [20], and *Locusta migratoria* [21]. Most of them such as the CPRs from *S. exigua* [19], *B. dorsalis* [18], *Aphis* (*Toxoptera*) *citricidus* (Kirkaldy) [22], *L. striatellus* [12], *Plutella xylostella* [23], and *L. migratoria* [21] have been found to be associated with metabolism and resistance to insecticides. Due to their possible role in insecticide detoxification, insect CPRs may represent possible molecular targets for new insecticides [5,12,16,21].

Little information is available on the function of the CPR gene in *S. litura* and whether silencing of this gene by RNA interference (RNAi) affects larval susceptibility to insecticides. RNAi is a powerful and widely used tool to down-regulate expression of specific genes in insects [24]. Previous studies in our laboratory showed that microinjection of double-stranded RNA (dsRNA) into *S. litura* larvae can effectively silence specific target genes [6,25].

In the present study, we cloned a full-length cDNA encoding CPR of *S. litura* (*SlCPR*). We used quantitative reverse transcriptase PCR (RT-qPCR) to analyze the *SlCPR* expression pattern at the insect’s different developmental stages and in various tissues prepared from fifth-instar larvae. To investigate whether *SlCPR* transcript levels affect the insect’s susceptibility to insecticides, *SlCPR*-silenced larvae were exposed to phoxim. The results showed increased mortality of *SlCPR*-silenced larvae as compared to control larvae.

## 2. Results

### 2.1. Cloning and Sequence Analysis of SlCPR

**Figure 1:**
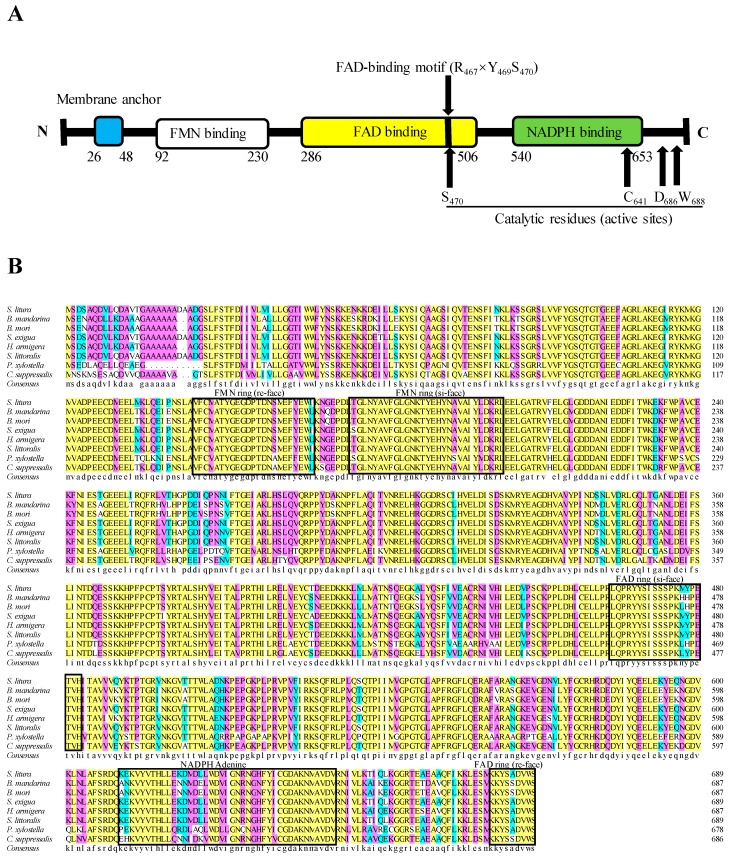
Sequence analysis of the *SlCPR* protein (**A**). The protein contains predicted flavin mononucleotide (FMN)-, flavin adenine dinucleotide (FAD)-, and NADP-binding domains. The proteins also conserved residues such as the FAD-binding motif (R467, Y469, and S470) and the predicted catalytic residues (S470, C641, D686, and W688). (**B**) Comparison of the deduced amino acid sequence of *SlCPR* with other NADPH-cytochrome P450 reductases (CPRs). Accession numbers of indicated CPR amino acid sequences are shown in Table 1.

In a previous study, a whole transcriptome analysis was performed for the midgut of *S. litura* fourth-instar larvae [25]. Based on these data, we identified and cloned a full-length *SlCPR* cDNA (GenBank Acc. MH638288). The *SlCPR* cDNA sequence contains a 237-bp 5′-untranslated region (5′-UTR), a 2070-bp open reading frame, and a 1783-bp 3′-UTR with a poly-A nucleotide sequence. The predicted protein contains 689 amino acids (77.72 kDa) and possesses a theoretical pI of 5.32. No signal peptide was identified at the N-terminus of the protein. However, a hydrophobic transmembrane region consisting of 22 amino acids was predicted (Figure 1). The three amino acid residues R467, Y469, and S470 constitute a putative FAD binding motif which is ubiquitous in the FAD binding domain of CPR proteins [26]. Similar to rat and other CPRs, conserved catalytic residues are present in the *SlCPR* protein (S470, C641, D686, and W688) (Figure 1A). These active site residues have been demonstrated to be essential for CPR activity [17,27]. According to Cheng et al. (2017) [13] and nucleotide sequences deposited at the DDBJ/ENA/GenBank databases, *S. litura* possesses a single copy of the *CPR* gene. The alignment results of the deduced amino acid sequences of *SlCPR* and some other known CPRs showed that *SlCPR* shared 99.6%, 98.4%, and 95.6% amino acid identity with the CPR sequences of *S. littoralis*, *S. exigua*, and *H. armigera*, respectively. The results also demonstrated that *SlCPR* is a new member of the CPR family (Figure 1B).

### 2.2. Phylogenetic Relation Between SlCPR and Other CPRs

**Figure 2:**
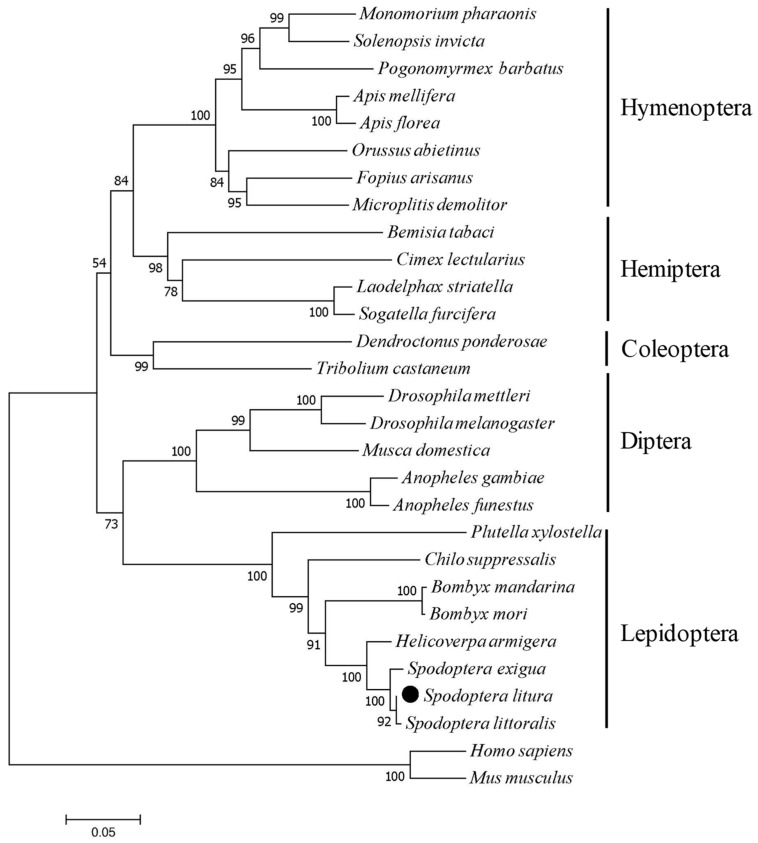
Phylogenic analysis of *SlCPR* and related insect CPRs. Multiple sequence alignment of full-length amino acid sequences of 29 CPR proteins was performed with DNAMAN 6.0 software. The phylogenetic tree was constructed using MEGA7.0 with the neighbor-joining (NJ) method and 1000 bootstrap replicates. Numbers shown at the tree forks indicate frequency of occurrence among all bootstrap iterations performed. The scale bar indicates 0.05 amino acid substitutions per site. CPRs from human and mouse were used as an outgroup. *SlCPR* is marked by a black solid circle. Accession numbers of indicated CPR amino acid sequences are shown in Table 1.

Based on the deduced amino acid sequence of *SlCPR* and 28 other CPRs, phylogenetic analysis was performed using MEGA 7.0 software and the neighbor joining method. The constructed tree showed that CPRs from insects of the same order were grouped together. As expected, *SlCPR* was most related to CPRs of other Lepidoptera insects, including *P. xylostella*, *Chilo suppressalis*, *Bombyx mandarina*, *Bombyx mori*, *H. armigera*, *S. exigua*, and *S. littoralis* (Figure 2, Table 1).

### 2.3. Developmental and Spatial Expression Patterns of SlCPR

**Figure 3:**
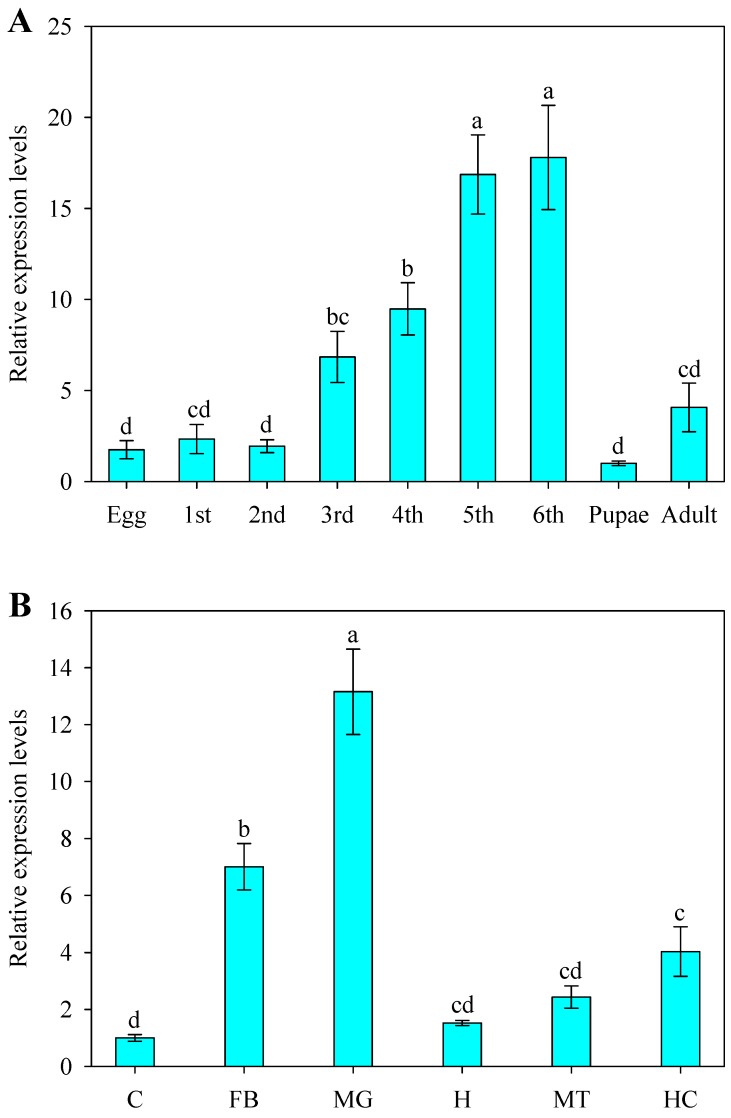
Relative expression levels of *SlCPR* at different development stages (**A**) and in various tissues (**B**). Whole body of *S. litura* larvae were used for the different development stages, while fifth-instar larvae were used for various tissues. Expression levels of *SlCPR* were determined by quantitative reverse transcriptase PCR (RT-qPCR), and *β-actin* and *EF1* were selected as reference genes. Each RT-qPCR reaction for each sample was performed in three biological replicates and three technical replicates. Data shown are means ± SE. Different letters (a,b,c,d) above bars indicate significant differences (*p* < 0.05) according to Duncan’s multiple range test. Abbreviations: 1st to 6th—first- to sixth-instar larvae; C—cuticle; FB—fat body; MG—midgut; H—head; MT—Malpighian tubule; HC—hemocytes.

We used RT-qPCR to examine the *SlCPR* expression pattern at different developmental stages of *S. litura*, namely eggs, first- to sixth-instar larvae, pupae, and adults. Highest expression levels were found in sixth-instar larvae (17.8-fold higher than in pupae), followed by fifth-instar larvae (16.9-fold higher than in pupae), and fifth-instar larvae (9.5-fold higher than in pupae) (Figure 3A). Tissue-specific expression of *SlCPR* was further analyzed for the cuticle, fat body, midgut, head, Malpighian tubule, and hemocytes of fifth-instar larvae (Figure 3B). Strongest expression levels of *SlCPR* were observed in the midgut (13.2-fold higher than in the cuticle) and fat body (7.0-fold higher than in the cuticle).

### 2.4. Expression Response of SlCPR in Larvae Exposed to Phoxim

**Figure 4:**
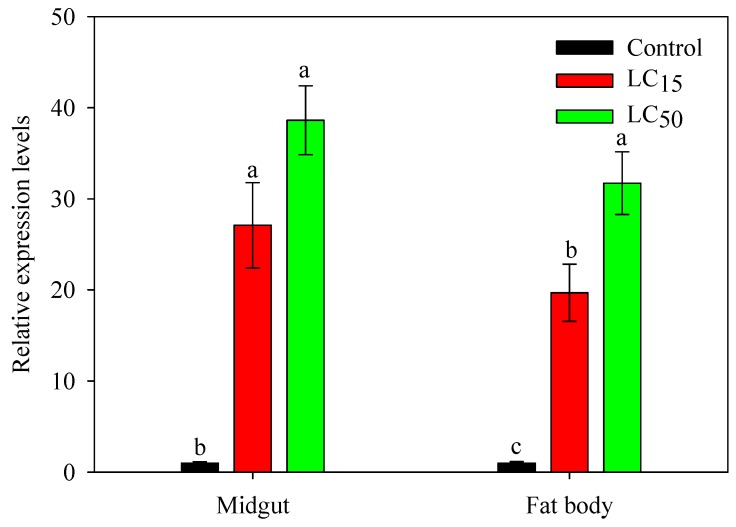
Effects of phoxim on *SlCPR* expression in the midgut and fat body of fifth-instar larvae. Larvae were exposed to phoxim at LC_15_ (4 μg/mL) and LC_50_ (20 μg/mL) doses for 24 h. *SlCPR* expression levels were normalized to *β-actin* and *EF1* expression and presented as the means ± SE with three independent biological replicates and three technical replicates. Different letters (a,b,c) above bars indicate significant differences (*p* < 0.05) according to Duncan’s multiple range test.

Phoxim was selected to examine insecticide effects on *SlCPR* expression in the midgut and fat body of fifth-instar larvae (Figure 4). Compared to control treatments, larvae exposed to phoxim at LC_15_ (4 μg/mL) and LC_50_ (20 μg/mL) doses showed significantly increased *SlCPR* expression levels in the midgut. At LC_50_, phoxim caused 38.6-fold increased expression of *SlCPR*. Similarly, exposure to phoxim significantly induced *SlCPR* expression levels in the fat body (19.6-fold increase at LC_15_ and 31.7-fold increase at LC_50_) (Figure 4).

### 2.5. Silencing of SlCPR by RNAi

**Figure 5:**
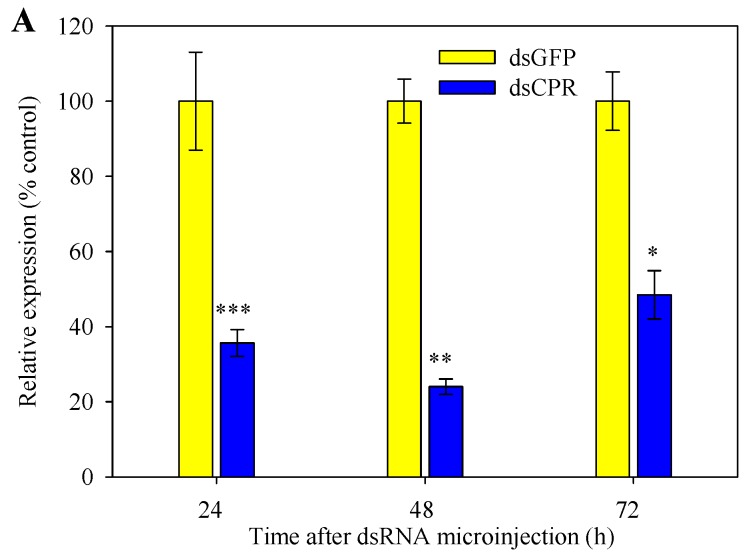

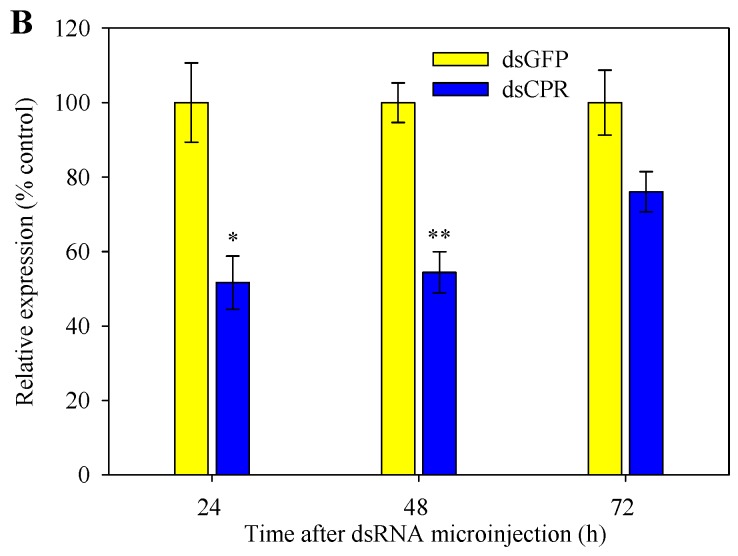
RNA interference (RNAi)-mediated silencing of *SlCPR* in fifth-instar larvae. Larvae were microinjected with dsCPR or dsGFP (control). RNA was isolated at indicated time points after microinjection. Expression levels of *SlCPR* in the midgut (**A**) and fat body (**B**) were then determined by RT-qPCR. The expression levels of *SlCPR* were normalized using *β-actin* and *EF1* as reference genes. Each RT-qPCR reaction for each sample was performed in three technical replicates and three biological replicates. Data indicate means ± SE. Asterisks indicate significantly reduced expression levels in *SlCPR*-silenced larvae as compared to the control group (Student’s *t*-test, * *p* < 0.05,** *p* < 0.01, *** *p* < 0.001).

RNAi-mediated silencing of *SlCPR* by dsCPR microinjection was performed with fifth-instar larvae. To determine the efficiency of silencing, expression levels of the *SlCPR*-silenced larvae were determined by RT-qPCR. Compared to control larvae that were microinjected with dsGFP, *SlCPR* expression in the midgut of *SlCPR*-silenced larvae significantly decreased by 64.3%, 76.0%, and 51.5% when analyzed at 24, 48, and 72 h after dsCPR microinjection (Figure 5A). Likewise, expression levels of *SlCPR*-silenced larvae in the fat body decreased after dsCPR microinjection (by 48.4% at 24 h; by 45.6% at 48 h) (Figure 5B). These results indicated that RNAi suppressed the expression of *SlCPR* in *S. litura* larvae and that the silencing effect was retained for at least 48 h.

### 2.6. SlCPR-Silenced Larvae Show Increased Susceptibility to Phoxim

**Figure 6:**
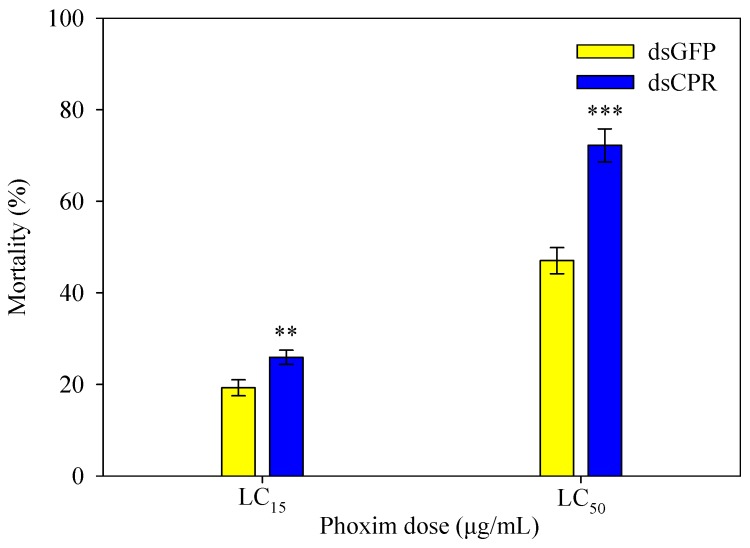
Effect of *SlCPR* silencing on the susceptibility to phoxim. Fourth-instar larvae were microinjected with dsCPR or dsGFP (control). Thirty fifth-instar larvae were then exposed to phoxim at LC_15_ (4 μg/mL) or LC_50_ (20 μg/mL) doses for 48 h. All tests were performed in triplicate. Data shown are mortality rates (means ± SE). Asterisks indicate significant differences between *SlCPR*-silenced larvae as compared to the control group (Student’s *t*-test, ** *p* < 0.01, *** *p* < 0.001).

Mortality rates of fifth-instar larvae that were first microinjected with dsCPR (or dsGFP) and then exposed to phoxim are shown in Figure 6. When larvae were injected with dsGFP, mortality was 19.3% at the LC_15_ dose and 47.0% at the LC_50_ dose, respectively. However, compared to these control larvae, phoxim-induced mortality of *SlCPR*-silenced larvae was considerably increased (by 34.6% at the LC_15_ dose; by 53.5% at the LC_50_ dose) (Figure 6). These results indicate that *SlCPR*-silenced larvae exhibit an increased susceptibility to phoxim.

## 3. Discussion

Insect CPRs in phylogenetic trees are clearly segregated into clusters that correspond to different insect orders [16,21]. In the present study, we cloned and characterized the *SlCPR* gene of *S. litura*. The amino acid sequence of *SlCPR* shares high similarity with known CPRs. Our phylogenetic analysis of CPRs indicated that the *SlCPR* was more closely related to the CPR of *S. littoralis* than to the CPR of *S. exigua.* Likewise, previous phylogenetic analysis indicated that the P450 protein CYP321A7 of *S. litura* is most similar to CYP321A12 of *S. littoralis* [25], suggesting a close genetic relationship between *S. litura* and *S. littoralis* detoxification genes. Sequence comparisons also indicated that *SlCPR* most probably contains a hydrophobic N-terminal transmembrane domain, suggesting that *SlCPR* is a membrane anchored protein. In general, location of CPRs at the endoplasmic reticulum membrane is considered as essential for CPR function [15,17,19]. In this way, co-localized partner P450s are provided with electrons [17,28]. Similar hydrophobic transmembrane regions have been predicted for CPRs in related species such as *S. exigua* [19], *C. lectularius* [15], *N. lugens* [17], *C. suppressalis* [29], *H. armigera* [16], and *L. migratoria* [21]. Multiple sequence alignment further indicated that the hydrophilic C-terminal domain of *SlCPR* likely possesses FMN-, FAD-, and NADP-binding domains that are conserved among CPRs of insects [17,26]. Furthermore, putative catalytic residues (S470, C641, D686, and W688), known to be indispensable for rat and human CPR [27,30] were identified in the *SlCPR* sequence. Taking these sequence properties together, they indicate that *SlCPR* is likely an enzymatically functional CPR.

We further used RT-qPCR to investigate the expression profile of *SlCPR* in *S. litura*. The results showed that the expression levels of *SlCPR* varied among different development stages and tissues. Expression of *SlCPR* was strongest in the fifth- and sixth-instar larvae and highest expression levels were determined for the midgut and fat body of fifth-instar larvae. These differences likely reflect different levels of CPR activity. The expression pattern of *SlCPR* was found to be similar to that of CPRs in other insects such as *N. lugens* (*NlCPR*) [17], *H. armigera* (*HaCPR*) [31], and *L. striatellus* (*LsCPR*) [12]. CPRs likely possess conserved functions in insects [22]. The observed expression profile of *SlCPR* suggests that the protein is associated with different co-expressed P450s required for detoxification of plant allelochemicals and/or insecticides.

Previous studies have shown that CPRs of insects (together with partner P450s) may play an important role in detoxification of plant allelochemicals and insecticides [5,16,19]. An upregulation of *CPR* expression in insects may increase their resistance to insecticides [17,20,32]. For example, expression levels of the *P. xylostella CPR* gene in fourth-instar larvae were 13.2-fold higher in a *β*-cypermathrin resistant strain than in a susceptible strain [23]. Likewise, as compared to an insecticide-susceptible strain, *CPR* expression levels in apterous adult *Rhopalosiphum padi* were higher in an isoprocarb-resistant strain and imidacloprid-resistant strain (by 3.74- and 3.52-fold, respectively) [32]. In the present study, *S. litura* larvae exposed to phoxim showed significantly increased *SlCPR* transcript levels in the midgut and fat body. These findings suggested that *SlCPR* could be involved in insecticide detoxification and prompted us to further examine *SlCPR*-silenced larvae for their susceptibility to phoxim. In fact, previous reports on various insects showed that microinjection or feeding of dsRNA can result in successful silencing of *CPR* genes and this may influence the insect’s susceptibility to insecticides [12,15,21]. When exposed to *β*-cypermethrin, the mortality rate of the of *NlCPR*-silenced third-instar nymphs of *N. lugens* was 59.5% whereas control nymphs (microinjected with dsGFP) showed only 26.2%. Imidacloprid showed similar effects in *NlCPR*-silenced nymphs [17]. Furthermore, increased susceptibility to carbaryl was observed for third-instar nymphs of *L. migratoria* silenced in *LmCPR* [21]. Moreover, in *A. citricidus*, silencing of *AcCPR* caused significantly increased mortality when the adult aphids were exposed to abamectin [22]. In the present study, we successfully silenced the expression of *SlCPR* in *S. litura* fifth-instar larvae. Expression levels in the midgut and fat body were significantly reduced after dsCPR microinjection. When exposed to phoxim at LC_15_ and LC_50_ doses, *SlCPR* silencing significantly increased the mortality of *S. litura* as compared to the control group microinjected with dsGFP. Hence, reduced *SlCPR* expression levels enhanced the susceptibility of *S. litura* larvae to phoxim. These results suggest that *SlCPR*, in combination with partner P450s, is implicated in detoxification of phoxim.

In conclusion, we provide in this study key information on the *SlCPR* gene and our data indicate that *SlCPR* expression levels in *S. litura* larvae influence their susceptibility to phoxim. Further studies are needed to identify the redox partners of *SlCPR* and to study their role in resistance of phoxim and other insecticides.

## 4. Material and Methods

### 4.1. Insects

The phoxim susceptible population of *S. litura* used in this study was originally obtained from the Insectarium of the Institute of Entomology, Sun Yat-sen University (Guangzhou, China, May 11 2017). *S. litura* larvae were fed on an artificial diet [33] and maintained in an insectary (without exposure to any insecticides for more than two years) at 25 ± 2 °C and 70% ± 5% relative humidity under a 16:8 h light:dark regime at South China Agricultural University (Guangzhou, China).

### 4.2. RNA Extraction and cDNA Synthesis

RNA was extracted from eggs (20 eggs per RNA extraction), first- to sixth-instar larvae at day 2 (three larvae per RNA extraction), pupae at day 2 (three pupae per RNA extraction) and adult at day 1 (three adults per RNA extraction) for analyses of the *SlCPR* expression pattern at different development stages of *S. litura*. RNA was extracted from various tissues (cuticle, fat body, midgut, head, Malpighian tubule, and hemocytes) of fifth-instar larvae for analysis of the *SlCPR* expression pattern in different tissue types. Hemocytes were obtained with microcapillaries according to previously described procedures [34,35]. The material was centrifuged (10,000× g, 4 °C, 10 min) to remove debris. To obtain fat body tissue, the midgut was opened with tweezers and the content was carefully removed. The white-yellow fat body was then scraped from the midgut with tweezers and transferred into an Eppendorf tube containing phosphate-buffered saline (PBS). The sample was then centrifuged (2000 rpm, 4 °C, 3 min) to remove PBS. Finally, the fat body was washed twice with PBS.

Tissues from three individuals were pooled to obtain one RNA sample. Three independent biological replicates were performed for all samples. The RNA extraction procedure was performed with the RNAiso Plus kit (TaKaRa, Dalian, China) following the manual instructions. Isolated RNA (1 μg) was reverse transcribed using the ThermoScript™ RT-PCR System kit (Thermo Fisher Scientific, Carlsbad, CA, USA) following the manufacturer’s instruction.

### 4.3. Cloning of SlCPR

Based on obtained *S. litura* transcriptome data [25], primers (*SlCPR*-full-F: 5′-ATGTCAGACAGCGCACAGGACGTTC-3′; *SlCPR*-full-R: 5′-ACTCCAAACGTCAGCAGAATATTTC-3′)) were designed to amplify the complete *SlCPR* gene. cDNA derived from RNA isolated from *S. litura* fourth-instar larvae served as template. The PCR product was purified (Qiagen PCR Purification Kit, Qiagen, Netherlands) and cloned into the pMD18-T vector (Takara, Dalian, China). Finally, the plasmid was transformed into *Escherichia coli* DH5α competent cells (Invitrogen, Carlsbad, CA, USA) following the supplier’s guidelines and sequenced. The full-length *SlCPR* sequence can be found in the GenBank database under the accession number MH638288.

### 4.4. Bioinformatic Analyses

The predicted molecular weight and isoelectric point of *SlCPR* were calculated using corresponding programs available at the ExPASy Proteomics Server (http://cn.expasy.org/tools/pi_tool.html). Signal peptide and subcellular localization predictions were made with the SignalP 3.0 (http://www.cbs.dtu.dk/services/SignalP/) and the WoLF PSORT (http://wolfpsort.org/) programs. Multiple sequence alignment of CPR amino acid sequences was performed with DNAMAN software package (Version 6.0, Lynnon Biosoft, Vaudreuil, Quebec, Canada) [36]. MEGA 7.0 software (MEGA, PA, USA) [37] was employed to construct a corresponding phylogenetic tree using the neighbor-joining method with 1000 bootstrap replicates.

### 4.5. SlCPR Expression Analysis

Relative expression levels of *SlCPR* were quantified by RT-qPCR, using obtained cDNA and *SlCPR* specific primers (*SlCPR-q*F: 5′-TTACATAAGGGTGGAGATAGG-3′; *SlCPR-q*R: 5′-TGGTCAGTGTTGATGAGAGAG-3′). The PCR product (185 bp in length) corresponded to the nucleotide position 913 to 1097 of the *SlCPR* coding region. Two reference genes, *β-actin* (GenBank Acc. No. DQ494753) and Elongation factor-1 (*EF1*) (GenBank Acc. No. DQ192234) were used for normalizing the target gene expression. We confirm that *β-actin* and *EF1* were the relatively stable genes for various target genes. The primers of the *β-actin* (*β*-actinF: 5′-TGAGACCTTCAACTCCCCCG-3′; *β*-actinR: 5′-GCGACCAGCCAAGTCCAGAC-3′) and *EF1* (*EF1*F: 5′-CTCCTACATCAAGAAGATC-3′; *EF1*R: 5′-CTTGAGGATACCAGTTTC-3′) have been used before [25,38]. Each RT-qPCR was performed in a 20-μL reaction volume that contained 10 ng of cDNA template, 10 μL SYBR Green I Master Mix (Roche Diagnostics Corp., Indianapolis, IN, USA) and 0.2 μM of each primer. Reactions were performed with a MJ Research Opticon^TM^ 2 instrument (Bio-Rad, Inc., Hercules, CA, USA) using the following parameters: (i) One cycle at 95 °C for 30 s and (ii) 40 cycles at 95 °C for 10 s and 60 °C for 25 s. The relative expression levels of *SlCPR* were calculated by the 2^−∆∆Ct^ method [39] and normalized to the two reference genes (*β-actin* and *EF1*). All RT-qPCR experiments were performed with three independent biological replicates.

### 4.6. Analysis of SlCPR Expression in Larvae Exposed to Phoxim

Phoxim (99.0%, Shanghai Jiang Lai Biotechnology Co., Ltd., Shanghai, China) was diluted in acetone (99.5%, Guangzhou Chemical Reagent Factory, China) to obtain a 100 μg/mL stock solution. Then, the stock solution was diluted with sterilized water to prepare different concentrations for the tests. Concentrations of phoxim causing 15% and 50% lethality of fifth-instar larvae (LC_15_: 4 μg/mL; LC_50_: 20 μg/mL) were used in this study. The LC_15_ and LC_50_ values were obtained from a trial experiment with different phoxim doses. The mortality values were 6.7%, 11.1%, 45.6%, 73.3%, 81.1%, and 96.7% at 1, 4, 16, 64, 256, and 1024 μg/mL phoxim, respectively (Figure S1). The LC_15_ and LC_50_ values were determined using probit analysis (POLO-PC software). To test toxicity of phoxim on *S. litura*, fifth-instar (day 1) larvae were used in a standard leaf disc bioassay method [1]. Leaves (7 cm in diameter) of Chinese cabbage (*Brassica campestris* L. ssp. pekinensis) were immersed in the prepared phoxim solution (LC_15_ or LC_50_ dosages) for 10 s and allowed to air-dry for 1.5 h. Control leaves were immersed in sterilized water. A total of 30 fifth-instar larvae were placed on each treated leaf (three larvae per leaf) which were placed in a sterile glass Petri dish (9 cm in diameter). After 24 h incubation in the insectary, the midgut or fat bodies from three of surviving larvae were pooled as one sample for RNA exaction, respectively. Three independent replicates were used for each treatment (three biological replicates). *SlCPR* expression analysis by RT-qPCR was conducted as described above.

### 4.7. Silencing of *SlCPR* by RNAi

DNA for in vitro transcription reactions was amplified by PCRs using cDNAs of *SlCPR* and *GFP* (green fluorescent protein; accession number ACY56286) as a control. The PCRs were performed with the following primers: (i) CPR-RNAi-F (5′-ATGGTTGCTGATCCCGAAGAA-3′) and T7CPR-RNAi-F (5′-aatacgactcactatagggATGGTTGCTGATCCCGAAGAA-3′), (ii) CPR-RNAi-R (5′-AGGCCAAACACGGCATAATTT-3′) and T7CPR-RNAi-R (5′-aatacgactcactataggg AGGCCAAACACGGCATAATTT-3′), (iii) T7GFPdsRNAF (5′-AATACGACTCACTATAGGGAAGGGCGAGGAGCTGTTCACCG-3′) and GFPdsRNAR (5′CAGCAGGACCATGTGATCGCGC-3′), and (iv) GFPdsRNAF (5′-AAGGGCGAGGAGCTGTTCACCG-3′) and T7GFPdsRNAR (5′-AATACGACTCACTATAGGGCAGCAGGACCATGTGATCGCGC-3′) [25]. The *SlCPR* PCR product corresponded to the nucleotide position 361 to 551 of the *SlCPR* coding region. The PCR products were then purified with a PCR purification kit (Qiagen, Venlo, The Netherlands) and used as templates to synthesize double-stranded RNA (dsRNA) with the T7 RiboMAX™ Express RNAi System (Promega, Madison, WI, USA). The dsRNA was adjusted with DEPC-treated (RNase-free) water to a final concentration of 1.5 μg·μL^−1^ and kept at –80 °C for further use. Subsequently, 2 μL (3.0 μg) of dsRNA were injected into the side of the thorax of fifth-instar (day 2) larvae of *S. litura* using a manual microinjector (model No. MS05, Chengdu Centome Company Ltd., Chengdu, China). Thirty fifth-instar larvae microinjected with dsCPR or dsGFP were incubated in the insectary for 24, 48, and 72 h, respectively. RNA was then isolated from the midgut and fat bodies, respectively. Tissue from three larvae were used for each RNA extraction. *SlCPR* expression levels in the midgut and fat bodies were determined by RT-qPCR. Three independent replicates were conducted for all treatments.

### 4.8. Bioassays with Phoxim after RNAi

To explore a possible role of *SlCPR* in the insect’s susceptibility to phoxim, dsCPR or dsGFP was microinjected into 30 fifth-instar (day 1) larvae of *S. litura*, respectively. Leaves of Chinese cabbage were immersed in phoxim solution (LC_15_ or LC_50_ doses) and then air-dried. After dsRNA delivery, *S. litura* were placed on each prepared leaf and incubated in the insectary at the same condition as described above. Mortality rates of *S. litura* were recorded after 48 h. All tests were performed in three independent replicates.

### 4.9. Data Analysis

Data were expressed as means ± standard error (SE). Statistical analysis was carried out with the SPSS 13.0 Software Package (SPSS Inc., Chicago, IL, USA). One-way ANOVA followed by the Duncan’s multiple range test was employed to analyze differences among different development stages and tissues. The Student’s *t*-test was used to analyze data from *SlCPR*-silenced larvae and toxicity tests with phoxim. Statistical differences were considered as significant at *p* < 0.05.

## Figures and Tables

**Table 1:** Percent amino acid identities between *SlCPR* and other CPRs.

Order	Species	Accession Number	Identity (%)
Hymenoptera	*Monomorium pharaonis*	XP_012541364	62.8
	*Solenopsis invicta*	XP_011157063	62.6
	*Pogonomyrmex barbatus*	XP_011643152	61.5
	*Apis mellifera*	XP_001119949	62.1
	*Apis florea*	NP_001351669	62.6
	*Orussus abietinus*	XP_012275162	62.6
	*Fopius arisanus*	XP_011306347	63.4
	*Microplitis demolitor*	XP_008548684	62.3
Hemiptera	*Bemisia tabaci*	AGT15701	61.9
	*Cimex lectularius*	AFD50507	62.7
	*Laodelphax striatella*	AID55422	63.8
	*Sogatella furcifera*	AHM93009	64.4
Coleoptera	*Dendroctonus ponderosae*	AFI45002	64.9
	*Tribolium castaneum*	XP_971174	67.6
Diptera	*Drosophila mettleri*	AAB48964	62.8
	*Drosophila melanogaster*	NP_477158	66.6
	*Musca domestica*	AAA29295	68.4
	*Anopheles gambiae*	AAO24765	66.6
	*Anopheles funestus*	EF152578	67.5
Lepidoptera	*Plutella xylostella*	NP_001292469	79.4
	*Chilo suppressalis*	AGM20565	85.4
	*Bombyx mandarina*	ABJ97709	87.0
	*Bombyx mori*	NP_001104834	87.0
	*Helicoverpa armigera*	ADK25060	95.6
	*Spodoptera exigua*	ADX95746	98.4
	*Spodoptera littoralis*	AFP20584	99.6
	*Spodoptera litura*	MH638288	100
Rodentia	*Mus musculus*	NM_008898	53.5
Primates	*Homo sapiens*	NP_000932	56.2

## Supplementary Materials

Supplementary materials can be found at https://www.mdpi.com/1422-0067/20/15/3839/s1.
