# Supplementary material for: Tobacco Cutworm (Spodoptera Litura) Larvae Silenced in the NADPH-Cytochrome P450 Reductase Gene Show Increased Susceptibility to Phoxim
Source: Int J Mol Sci. 2019 Aug 6;20(15):3839. doi: 10.3390/ijms20153839 (PMC6696589; doi:10.3390/ijms20153839)
Supplement: Supplementary file 1 [file ijms-20-03839-s001.pdf]

SUPPLEMENTARY INFORMATION

Tobacco cutworm (*Spodoptera litura*) larvae silenced in the NADPH-cytochrome P450 reductase gene show increased susceptibility to phoxim

Hong-Yi Ji, Christian Staehelin, Yan-Ping Jiang, Shi-Wei Liu, Zhi-Hui Ma, Yi-Juan Su, Jia-En Zhang, and Rui-Long Wang

Int. J. Mol. Sci. 2019

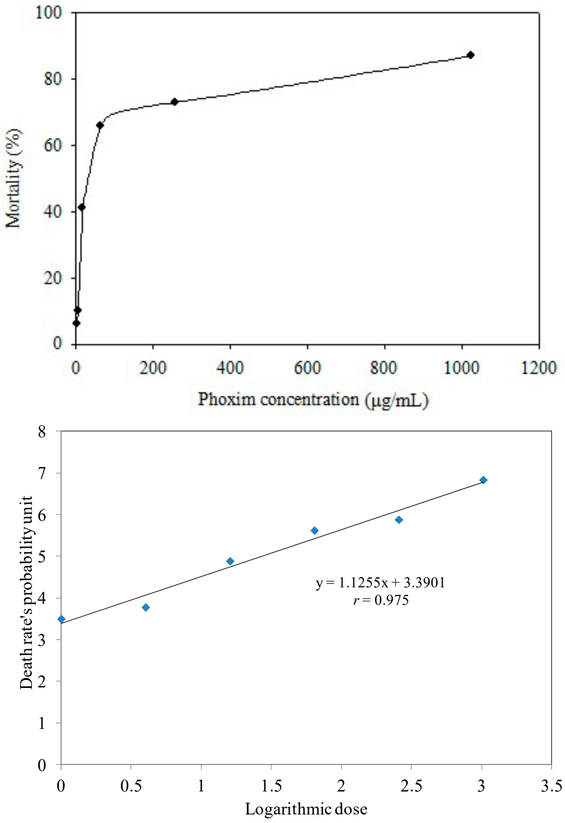

Supplementary Figure 1: Determination of LC_{15} and LC_{50} values for phoxim. Fifth-instar larvae of *S. litura* were treated with indicated concentrations of phoxim and mortality values were determined 48 hours after the insecticide treatment. The upper panel indicates dose-dependent mortality and the lower panel shows the linear regression equation after logarithmic transformation.
